# Verbal and non-verbal semantic impairment: From fluent primary progressive aphasia to semantic dementia

**DOI:** 10.1590/s1980-57642008dn10200014

**Published:** 2007

**Authors:** Mirna Lie Hosogi Senaha, Paulo Caramelli, Claudia Sellitto Porto, Ricardo Nitrini

**Affiliations:** 1 PhD. Member of Behavioral and Cognitive Neurology Unit of the Department of Neurology, University of São Paulo School of Medicine, São Paulo, Brazil.; 2 MD, PhD. Associate Professor, Department of Internal Medicine, Faculty of Medicine, Federal University of Minas Gerais, Belo Horizonte, Minas Gerais, Brazil.; 3 PhD. Member of Behavioral and Cognitive Neurology Unit of the Department of Neurology, University of São Paulo School of Medicine, São Paulo, Brazil.; 4 MD, PhD. Associate Professor, Department of Neurology, University of São Paulo School of Medicine, São Paulo, Brazil.

**Keywords:** semantic dementia, semantic memory, temporal lobe, progressive primary aphasia, semantic system

## Abstract

Selective disturbances of semantic memory have attracted the interest of many investigators and the question of the existence of single or multiple semantic systems remains a very controversial theme in the literature.

**Objectives:**

To discuss the question of multiple semantic systems based on a longitudinal study of a patient who presented semantic dementia from fluent primary progressive aphasia.

**Methods:**

A 66 year-old woman with selective impairment of semantic memory was examined on two occasions, undergoing neuropsychological and language evaluations, the results of which were compared to those of three paired control individuals.

**Results:**

In the first evaluation, physical examination was normal and the score on the Mini-Mental State Examination was 26. Language evaluation revealed fluent speech, anomia, disturbance in word comprehension, preservation of the syntactic and phonological aspects of the language, besides surface dyslexia and dysgraphia. Autobiographical and episodic memories were relatively preserved. In semantic memory tests, the following dissociation was found: disturbance of verbal semantic memory with preservation of non-verbal semantic memory. Magnetic resonance of the brain revealed marked atrophy of the left anterior temporal lobe. After 14 months, the difficulties in verbal semantic memory had become more severe and the semantic disturbance, limited initially to the linguistic sphere, had worsened to involve non-verbal domains.

**Conclusions:**

Given the dissociation found in the first examination, we believe there is sufficient clinical evidence to refute the existence of a unitary semantic system.

## Abstract (pt)

Distúrbios seletivos da memória semântica têm atraído o interesse de muitos pesquisadores e a questão da existência de sistema semântico único ou múltiplo permanece como um tema muito controvertido na literatura.

**Objetivos:**

Discutir a questão de sistemas semânticos múltiplos por meio de um estudo longitudinal de uma paciente com afasia progressiva primária fluente que evoluiu para um quadro de demência semântica.

**Métodos:**

Uma mulher de 66 anos com comprometimento seletivo da memória semântica foi avaliada em dois momentos por meio de testes neuropsicológicos e fonoaudiológicos e os resultados foram comparados aos de três indivíduos controles pareados.

**Resultados:**

Na primeira avaliação, o exame físico estava normal e a paciente obteve 26 pontos no Mini-exame do estado mental. Na avaliação da linguagem foram constatadas: produção oral fluente, anomia, comprometimento da compreensão de palavras, preservação dos aspectos sintáticos e fonológicos da linguagem e dislexia e disgrafia de superfície. As memórias autobiográfica e episódica encontravam-se relativamente preservadas. Em testes de memória semântica, a seguinte dissociação foi constatada: distúrbio da memória semântica verbal com preservação da memória semântica não-verbal. Os exames de ressonância magnética cerebral revelaram atrofia acentuada do lobo temporal anterior do hemisfério cerebral esquerdo. Após 14 meses, as dificuldades da memória semântica verbal tornaram-se mais intensas e o distúrbio semântico, inicialmente restrito, à esfera verbal passou a domínios não-verbais.

**Conclusões:**

Dada a dissociação encontrada na primeira avaliação, acreditamos que temos evidências clínicas contra a existência de um único sistema semântico.

In 1992, five cases of semantic dementia (SD) were described by Hodges et al.^1^ who distinguished this type of fluent progressive aphasia from the non-fluent primary progressive aphasia described initially by Mesulam.^2^ Hodges et al.^1^ proposed the following criteria for the diagnosis of this syndrome:

(1) selective impairment of semantic memory causing severe anomia, impaired spoken and written single-word comprehension, reduced generation of exemplars on category fluency tests and an impoverished general knowledge;
(2) relative sparing of other components of language, notably syntax and phonology;
(3) normal perceptual skills and non-verbal problem-solving abilities;
(4) relatively preserved autobiographical and episodic memory;
(5) surface dyslexia.

In SD, the intense vocabulary loss associated with the difficulty in recognizing pictures, objects or famous people’s faces, in the absence of perceptive alterations, could be taken as evidence of the existence of a single semantic system. The existence of single or multiple semantic systems remains a very controversial theme in the literature.^3-11^ Caramazza et al.^5^ and Hillis et al.^6^ supported the hypothesis of a single semantic system and proposed a model of semantic processing called OUCH (Organized Unitary Content Hypothesis). On the other hand, Shallice^12-14^ advocates modularity of the semantic system, making a separation between the visual semantic system and verbal semantic system. He refers to modality-specific aphasias, disturbances of modality-specific semantic memory and effects of modality-specific priming.

The objectives of the current investigation were to explore and discuss the question of multiple semantic systems based on a case-study of a patient with a semantic memory disturbance.

## Methods

### Subjects

EHO, a 66 year-old retired Biology teacher, presented in 1998 with a two-year history of progressive word-finding difficulties and impaired single word comprehension. Her autobiographical memory and her daily life activities were unaffected and she continued running the household and engaging in voluntary work, which entailed the accounting of a charity home for children. Her behavior and personality were reported as unchanged.

**Figure 1:**
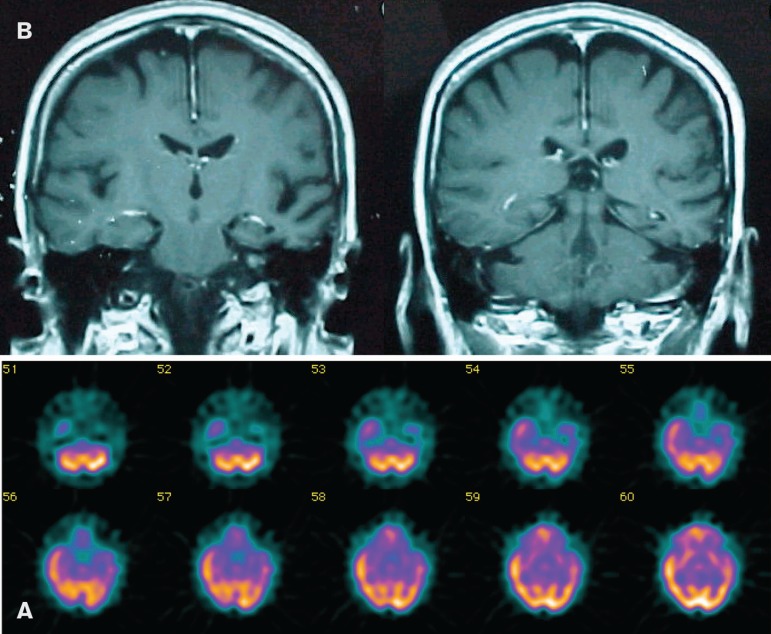
(A) Magnetic resonance imaging (MRI) showing atrophy of the left anterior temporal lobe; (B) Brain SPECT showing hypoperfusion in the left anterior temporal lobe

Physical exam, performed in 1998, was normal and the score in the Mini-Mental State Examination^15-16^ was 26. Routine laboratory tests and electroencephalogram were also normal. Resonance imaging revealed left temporal lobe atrophy, and brain SPECT showed left anterior temporal lobe hypoperfusion (Fig 1).

EHO’s performances in several tasks in neuropsychological and language assessments were impaired due to her language difficulties (Table 1). In spontaneous speech, EHO’s oral production was fluent, but marked by anomia. Phonological and syntactic aspects of oral language were preserved. Sentence comprehension was appropriate. However, oral and written word comprehension was impaired. Her greatest difficulty was in naming tasks.

**Table 1:** EHO's performances in neuropsychological and language evaluation.

	Results / Correct	
Tests	responses	
**MMSE**	26/30	
**Dementia rating scale**		
Attention	35/37	(0.16 SD*)
Initiation/perseveration	29/37	(-1.12 SD*)
Construction	6/6	(0.30 SD*)
Conceptualization	26/39	(-1.89 SD*)
Memory	20/25	(0.98 SD*)
Total	116/144	(-1.74 SD*)
**WAIS**		
**Verbal**		
Information	3	(-2.01 SD)
Comprehension	7	(-0.83 SD)
Arithmetic	10	(0.21 SD)
Similarities	9	(0.08 SD)
Digit span	9	(0.30 SD)
Vocabulary	7	(-0.89 SD)
Digit symbol	13	(3.15 SD)
** Performance**		
Picture completion	10	(0.60 SD)
Block design	13	(2.08 SD)
Object assembly	10	(1.09 SD)
Picture arrangement	14	(2.80 SD)
** Verbal IQ**	91	(-0.9 SD)
** Performance IQ**	133	(3.3 SD)
** Total IQ**	110	(1.0 SD)
**Trail making test**		
Part A	33s	P 50
Part B	131s	P 25
**Rey-Osterrieth complex figure**		
Copy	29/36	(-0.42 SD)
Memory	9/36	(-0.69 SD)
**Logical memory (WMS)**	1.5/23	(-2.25 SD)
Immediate recall	1.5/23	(-1.58 SD)
Delayed recall (30')		
**Visual reproduction (WMS)**	10/14	(1.09 SD)
Immediate recall	10/14	(1.40 SD)
Delayed recall (30')		
**Paired associates learning test (WMS)**	7/21	(-2.89 SD)
Immediate recall	4/7	(-2.66 SD)
Delayed recall (30')		
**Language comprehension tasks**		
Oral word comprehension (Beta)	13/17	
Written word comprehension (Beta)	10/13	
Oral sentence comprehension (Beta)	38/38	
Written sentence comprehension (Beta)	8/8	
Token Test - 1^st^ part	10/10	
Token Test - 2^nd^ part	6/6	
Token Test - 3^rd^ part	9/10	
Token Test- 4^th^ part	9/10	
Token Test - 5^th^ part	14/21	
Token Test - 5^th^ part (adapted version)**	19/21	
**Language production tasks**		
Boston naming test	10/60	
Reading aloud of words and non-	26/30	
words (Beta)		
Word and non-word dictation (Beta)	9/10	
Repetition of words and non-words	30/30	
(Beta)		
Repetition of sentences (Boston)	13/16	

MMSE, mini-mental status examination; DRS, dementia rating scale; WAIS, Wechsler adult intelligence scale; WMS, Wechsler memory scale; SD, standard deviation; P, percentile;

* according to Brazilian version^21^;

** adapted version of Token Test - 5^th^ part: the word "touch" was substituted by "lean".

Three control individuals, right-handed women aged 60 to 65, all graduated in education as EHO, were also evaluated.

## Materials and procedures

Matching, sorting, lexical decision, naming and other tasks were carried out. Some of these were adapted from the semantic memory protocol,^1,17^ while others were specifically devised to characterize EHO’s disturbance. A re-evaluation was performed 14 months after the first examination.

For comparative purposes, the tasks were also applied to the three control individuals.

Statistical analysis was conducted, through Fisher's exact test. The value of significance accepted was 0.05.

To facilitate the comprehension of the investigations the data were divided into three test series.

### TEST SERIES 1: SEMANTIC MEMORY EVALUATION

#### 1. Naming and verbal fluency

*1.1. Oral naming* – The oral naming of the 90 pictures adapted from the semantic memory protocol^1,17^ was requested. The pictures represented thirteen semantic categories.

*1.2. Written naming* – The written naming of the same pictures was requested.

*1.3. Verbal fluency*^1,17^ – Category and letter-based fluency (FAS) tests were performed.

#### 2. Matching

*2.1. Oral word-picture matching* – In this task, the examiner said a word and the individuals had to indicate the picture corresponding to this word, picking from eight pictures in the same semantic category. This task comprised 90 target stimuli.

*2.2. Written word-picture matching* – The task above was adapted, whereby all the presented oral words were converted to written words.

*2.3. Sound-picture matching* – Twenty-two sounds relating to non-verbal sounds such as musical instruments and nature sounds were presented separately and the individuals had to select, from four pictures, the drawing corresponding to the sound.

*2.4. Visual semantic matching (Protocole d’évaluation des gnosies visuelles - Agniel)*^18^ – The individuals had to associate a target picture to another which was semantically related, choosing from three alternatives (example: padlock - key/rose/bowl).

*2.5. Verbal semantic matching* – The task above was adapted, whereby all the presented visual stimuli were converted to their verbal forms.

#### 3. Sorting

*3.1. Visual sorting*^1,17^ – The picture sorting was requested at three different levels:

– Level I: classifying 48 pictures into living or inanimate items.
– Level II: (a) classifying the living pictures into land, aquatic animals or birds. (b) classifying the inanimate items into household items, musical instruments or vehicles.
– Level III: (a) classifying the land animal pictures according to habitat, size and ferocity was requested. (b) classifying the household items according to size, kitchen and electrical or non-electrical objects.

*3.2. Verbal sorting* – The task above was adapted, so that all the presented pictures were converted into their verbal form.

#### 4. Gesture production

In this task the individuals were asked to make gestures from pictures.

#### 5. Reading and dictation

The reading and dictation from the HFSP protocol^19^ were applied.

### TEST SERIES 2: INPUT LEXICON AND VISUAL REPRESENTATION EVALUATION

#### 1. Object decision

In this task, 24 pictures were employed, twelve corresponding to concrete/real images, and twelve pictures with no correspondence to any real item. Each picture was presented in a random order and the individuals had to state whether the picture corresponded to something real or not.

#### 2. Face recognition

Thirty faces, fifteen famous people and fifteen unknown faces, were used. Each picture was presented, in a random order whereby the individuals had to say if the person was famous or not.

#### 3. Oral lexical decision

Thirty two oral stimuli were utilized, where sixteen stimuli referred to real words while the other half corresponded to non-words based on the real words while keeping the same structure and syllabic length as the originals. Each stimulus was presented orally in random order. The individuals were asked to indicate whether the stimulus corresponded to a real word or not.

#### 4. Orthographic lexical decision

This task comprised 28 written stimuli, with half corresponding to real words and half to non-words. Each stimulus was presented, in random order, and the individuals had to state whether the written stimulus corresponded to a real word or not.

#### 5. Homophonic heterographic written words definition

This task was composed of twelve pairs of written words. Each pair comprised two homophonic heterographic words (words with different spelling but presenting the same pronunciation) such as, “cesta” (basket) and “sexta” (Friday) in Portuguese, or “mail” and “male” in English. The written words were presented in random order and individuals were then requested to provide the meaning of the words. The goal of this task was to investigate access from the input logographic lexicon to the semantic system. The integrity of this access is verified in the absence of confusing meanings between homophonous words.

### TEST SERIES 3: RE-EVALUATION

In re-evaluation, the matching, sorting and naming tasks described in test series 1 were reapplied and the following new tasks were performed:

#### 1. Tactile semantic matching

This task was composed of 20 target stimuli each, having five alternatives. The target stimulus was an object concealed inside a bag which the individuals had to touch, without seeing it. After touching, the individuals were requested to match the object touched, with one of five objects placed on the table which presented some semantic relationship to the target, for example, key/lock.

#### 2. Orthographic semantic matching

The task above was adapted, so that all the presented objects were converted into their written words.

#### 3. Object and tactile naming

The oral naming of the 20 objects was requested in two different ways. Firstly, the individuals had to name objects by touch without seeing them. Secondly, oral naming upon seeing the same objects was requested.

## Results

The low number of items produced by EHO in verbal fluency coupled with her performance in naming tasks, demonstrate her semantic-lexical difficulty (Table 2). Moreover, it was verified that EHO produced a larger number of items in FAS fluency than in category fluency.

**Table 2:** EHO's performance in verbal fluency and naming tasks in the first evaluation.

		Control individuals	
Tasks	EHO	mean	p
Verbal fluency			
**Semantic category (living things)**			
Land animals	4	19.3	-
Aquatic animals	2	13.3	-
Birds	1	11.0	-
Breeds of dog	0	6.0	-
Total-living things	7	49.6	-
**Semantic category (artifacts)**			
Household items	7	19.0	-
Vehicles	4	12.7	-
Musical instruments	1	13.7	-
Types of boats	1	5.0	-
Total - artifacts	13	50.3	-
**FAS**			
F	11	18.7	-
A	7	13.7	-
S	7	15.0	-
Total - F, A, S	25	47.3	-
**Oral naming**	41/90	84/90	<0.001*
**Written naming**	35/90	84/90	<0.001*

* significant at 0.05 level.

In both matching and sorting tasks, EHO’s poorest performance occurred when the input of information was through linguistic stimuli (Table 3). For non-linguistic stimuli, non-verbal sounds and pictures, EHO's performances were similar to those of the controls.

**Table 3:** EHO's performance in matching and sorting tasks in the first evaluation.

		% success		
			Control individuals	
Tasks	n	EHO	mean	p
**Matching (linguistic stimuli)**				
Oral word-picture	90	82.2	99.6	<0.001*
Written word -picture	90	82.2	100.0	<0.001*
Verbal semantic	20	80.0	98.3	0.013*
**Matching (non-linguistic stimuli)**				
Sound-picture	22	90.9	90.9	1
Visual semantic	20	100.0	100.0	1
**Verbal sorting (linguistic stimuli)**				
Level 1 (living; inanimate)	48	89.6	100.0	0.001*
Level 2 a (land; aquatic animal; bird)	24	75.0	100.0	<0.001*
Level 2 b (household; vehicles; music.)	24	83.3	95.8	0.063
Level 3 a (ferocity; size; habitat)	36	61.1	93.5	<0.001*
Level 3 b (kitchen; size; electrical)	36	88.9	99.1	0.014
**Visual sorting (non-linguistic stimuli)**				
Level 1 (living; inanimate)	48	100.0	100.0	1
Level 2 a (land; aquatic animal; bird)	24	100.0	98.6	1
Level 2 b (household; vehicles; music.)	24	100.0	95.8	0.571
Level 3 a (ferocity; size; habitat)	36	97.2	90.7	0.292
Level 3 b (kitchen; size; electrical)	36	100.0	99.1	1

* significant at 0.05 level.

In the gesture production from pictures task, the patient and controls managed to produce appropriate gestures for all items.

When comparing EHO's performance in the oral word-picture, written word-picture matching and naming tasks, we observed a close relationship between errors committed across the different tests. All the items that resulted in failures on the oral comprehension task were left unnamed by EHO in the subsequent naming task. Similarly, all the inadequate responses in the written comprehension task were on items that were also not named.

In reading, EHO’s performance ranged from 90% to 100%, on lists of non-words, regular and close class words. Her performance was not influenced by linguistic variables such as frequency, length and imageability. On the other hand, EHO read only 67.5% of the irregular words correctly, while a similar pattern was observed in writing upon dictation. EHO displayed good performance in writing non-words, regular and closed class words; 80%, 100% and 95%, respectively. For irregular words however, EHO wrote only 56.7% of the items correctly. These data and qualitative analyses showing a large number of regularizations, suggested that EHO presented surface dyslexia and dysgraphia.

In most of the tests in series 2, lexical, object decision and face recognition tasks, EHO achieved satisfactory performances as displayed in Table 4. EHO's performances in these tasks reveal the integrity of the input lexicons (logographic and logophonic) and also of the input representations involving visual-figurative stimuli and faces.

**Table 4:** EHO's performance in decision tasks in the first evaluation.

		% success		
			Control individuals	
Decision tasks	n	EHO	mean	p
Object decision	24	95.8	93.1	1
Face recognition	30	90.0	91.1	1
Oral lexical decision	32	100.0	93.7	0.336
Orthographic lexical decision	28	92.8	95.2	0.639

These data suggestive of input lexicon preservation are reinforced by EHO's performance in word definition. In some words for which the patient did not provide the appropriate definition, she stated that the words were not totally unknown to her, but that she was unable to access their meaning.

In 12 word pairs of homophonic heterographic written words definition, EHO provided the correct meaning for 14 (62,5%), whereas the average of controls was 86.1% (Table 5).

**Table 5:** EHO's performance in homophonic heterographic written words definition task in the firs evaluation.

Homophonic heterographic written definition task	EHO	Control individuals mean	p
Correct responses	14/24	20.7/24	0.007*
Absence of response in pointing out graphic form of word	4	0.7	0.033*
Absence of response	1	1.0	1
Indefinite mistakes (without apparent relationship with the target)	3	0	0.234
Supply of the meaning of the homophonic and heterographic word	2	1.7	0.416

* significant at 0.05 level.

Comparing EHO's responses to the controls showed that EHO presented a larger number of mistakes, probably as a consequence of semantic memory disturbance, given half of the mistakes were due to the absence of response. On the other hand, considering solely the confusing meanings between heterographic homophonic written words, EHO made two mistakes. Control 1 made two mistakes, control 2 made none while control 3 made three mistakes. These data suggest that EHO did not have impairment in access from the input logographic lexicon to the semantic memory.

**Figure 2:**
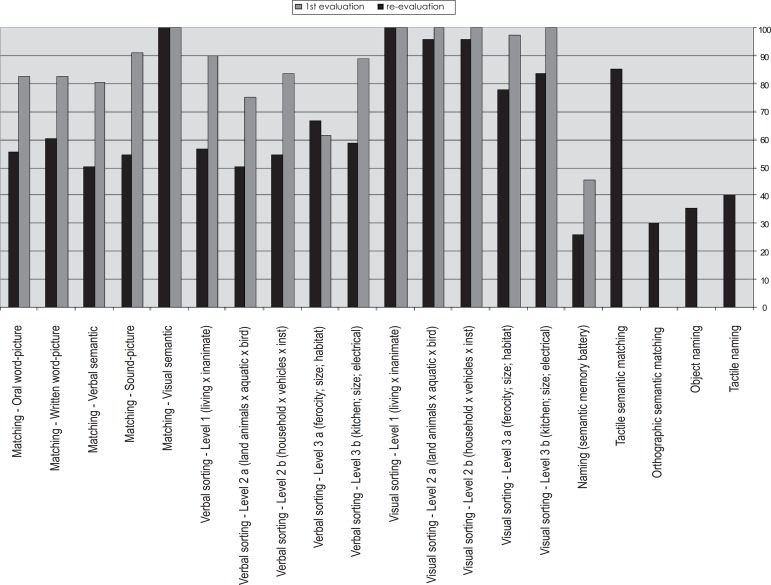
EHO's performance (%) in tasks at first evaluation and re-evaluation.

In re-evaluation, EHO’s performances in matching tasks fell, except in the visual semantic matching task (Fig 2). These falls occurred in all tasks involving linguistic items – oral word-picture, written word-picture, verbal semantic matching – and in the sound-picture matching task. In the first evaluation on the sorting tasks, an excellent performance by EHO was observed in those tasks involving picture stimuli, in contrast to the impaired performance in the classification of verbal stimuli. Upon re-evaluation, the dissociation between visual and verbal sorting was still evident; however, EHO made some errors in the visual sorting tests, suggesting an initial disturbance in visual semantic memory.

Responses indicative of disturbance in the visual semantic memory were also seen in oral naming tasks. During the first evaluation, EHO did not commit errors related to difficulties in visual semantic knowledge of pictures. However, this mistake type was encountered in the re-evaluation, albeit in small number. For example, in the *lock* picture during the first examination, EHO said: “to put a key in, isn’t it? But it has a name...” and in re-evaluation she said: “what is this? I have no idea”. In the *asparagus* picture in the first evaluation, EHO said: “it looks like that thing to make salad. Oregano?” and in the re-evaluation, her response was: “what is this? An animal? Here is the mouth, but I don’t know what it is”.

During re-evaluation, in the two new semantic matching tasks - one from graphic and another from tactile stimuli - the dissociation between the performances based on linguistic and non-linguistic stimuli were clear. In the new naming tasks the performances were independent of the input modality of the stimulus being named.

## Discussion

EHO's difficulties in oral and written word comprehension, oral and written naming, reading and writing of irregular words can be interpreted as indicative of a selective impairment in semantic memory. To support the hypothesis of the isolated impairment of the semantic system, tasks were designed to examine the integrity of the input lexicon – logographic and logophonic – and the input visual representations. The results suggest these lexicons integrity. It was possible to test access from the input logographic lexicon to the semantic system by the homophonic heterographic written words definition. In this task EHO made a few mistakes – as did the controls – related to confused meaning between homophonic heterographic words, thus suggesting the integrity of this access. The other accesses, from the input logophonic lexicon to the semantic system, and from the visual representation to the semantic system, are difficult to test separately. Therefore, the available data points to the notion that EHO’s disturbance is characterized by semantic degradation with preservation of input lexicons. Moreover, in the visual and verbal stimuli sorting tasks, we observed that the mistakes occurred only in verbal sorting tasks, while her performance in the visual sorting task was excellent, even for the more specific items.

According to the models of lexical processing allowing for the existence of a single semantic system, as in the model by Hillis,^20^ a functional disturbance of the semantic memory would also impair the semantic comprehension of pictures, objects and non-verbal sounds. However, no alterations were observed in any tasks involving the comprehension of non-verbal stimuli during EHO’s first evaluation (visual semantic matching, sound-picture matching, production of gestures based on pictures and visual sorting). In the production of gestures task, EHO presented a good performance, as well as in the sound-picture matching task. Also, in the oral naming task, no mistakes resulting from the visual semantic knowledge of pictures were seen.

How can this be explained? According to the single semantic system models, the pathological process should have affected the stimuli comprehension, regardless of the input modality. The single semantic system models do not explain the dissociation found in EHO’s first evaluation, namely: excellent performance in tasks related to non-verbal semantic memory in conjunction with difficulties in tasks related to verbal semantic memory. In the gesture production task, EHO was able to perform the gestures accurately. However, according to Caramazza et al.,^5^ the accurate production of gestures based on pictures does not imply the existence of multiple semantic systems, because sometimes it is not possible to know if the gesture was properly performed. How can one distinguish between the gestures representative of a truck or a car, and between tea or coffee?

By the same token, how can EHO's performances in the sorting tasks be explained? EHO experienced difficulties in the verbal sorting, but had satisfactory performance in visual classification tasks. Some authors, such as Caramazza et al.,^5^ support the idea that figurative representation already carries implicit information on meaning. Something that has feet or eyes is probably a living thing. We believe that only further information of a generic nature can be extracted from figurative stimuli, such as classifying a picture into living or inanimate, as required in the sorting tasks (level 1). Analyzing the visual sorting tasks that we adapted from the semantic memory protocol,^1,17^ sorting level 2 requests the classification of living things into: land, aquatic animals, and birds, whilst the objects into: domestic utensils, musical instruments and transport. At this level, some information in the drawing can indeed facilitate the classification; for instance, the existence of wheels suggests that it is a vehicle, the existence of wings indicates that the animal flies, and so on. In the last part of the task - sorting level 3 - the requested response no longer depends on the visual information taken from the figurative stimulus. For example, on visual sorting (level 3) the individuals have to decide if the picture of a bear is larger or smaller than a man, whether the animal is native to Brazil or not, and whether it is ferocious or not. In other words, the information required on classification by size, habitat and ferocity is not given away by any visual picture characteristics. The household items have to be classified into: size, whether they are used in the kitchen or not and if they are electrical or otherwise. In the same way, the visual characteristics of the pictures do not give away any information to help accomplish this sorting.

In the visual sorting task - level 3, EHO also presented good performance, reinforcing the existence of a dissociation in the sorting tasks: EHO's performance in visual classification was similar to the controls, whereas verbal sorting was highly impaired, thereby suggesting the existence of at least one visual semantic and one verbal semantic system. Moreover, EHO demonstrated similar performance to the controls in the sound-picture matching task. In this task no verbal stimulus is used, while it is considered by Bozeat et al.^3^ to be sensitive in detecting mild semantic problems.

In conclusion, given the dissociation found in the first examination on the sorting tasks, the good performance in sound-picture matching, the integrity of the input lexical memories and the preservation of the access from the input logographic lexicon to the semantic system, we believe that sufficient clinical evidence can be drawn from this case study to refute the existence of a unitary semantic system.

One of the characteristics mentioned by Hodges et al.^1^ to diagnose SD - impoverishment of general knowledge - was not found in our patient upon first evaluation. After 14 months, however, the difficulties in verbal semantic memory had increased. In addition to this impaired function, other difficulties in visual sorting of figurative stimuli, mistakes in naming for non-semantic recognition of pictures, and difficulties in the sound-picture matching, hitherto not seen in the first evaluation, were later present. These data suggest that the semantic disturbance of EHO, limited initially to the linguistic domain, had spread to other, non-verbal domains. Moreover, in the course of the disease, symptoms related to frontal lobe dysfunction, such as disinhibition and childish behavior emerged. In other words, the patient’s picture, characterized initially by selective disturbance of verbal semantics expanded to become widespread semantic impairment and behavioral changes. Such characteristics can be associated with the diagnosis of SD. The selective disturbance of verbal semantic memory could be the initial symptom of SD in the case of EHO.
